# Trends in risk classification and primary therapy of Japanese patients with prostate cancer in Nara urological research and treatment group (NURTG) – comparison between 2004–2006, 2007–2009, and 2010–2012

**DOI:** 10.1186/s12885-017-3637-2

**Published:** 2017-09-02

**Authors:** Nobumichi Tanaka, Yasushi Nakai, Makito Miyake, Satoshi Anai, Takeshi Inoue, Tomomi Fujii, Noboru Konishi, Kiyohide Fujimoto

**Affiliations:** 1 Department of Urology, Nara Medical University, Nara, Japan; 2 Department of Pathology, Nara Medical University, Nara, Japan

**Keywords:** Primary therapy, Primary androgen deprivation therapy, Radical prostatectomy, Radiation therapy, Risk classification, Active surveillance

## Abstract

**Background:**

To assess the trends in risk classification and primary therapy of Japanese prostate cancer patients who were diagnosed between 2004 and 2012.

**Methods:**

A total of 7768 patients who were newly diagnosed with prostate cancer at Nara Medical University and its 23 affiliated hospitals between 2004 and 2012 were enrolled. The trends in risk classification and primary therapy in 2004–2006 (prior period), 2007–2009 (middle period), and 2010–2012 (latter period) were compared.

**Results:**

The proportion of high-risk and worse patients significantly decreased in the latter period compared to the prior period (*p* < 0.001), while that of intermediate-risk patients significantly increased over the years (*p* < 0.001). The proportion of primary androgen deprivation therapy (PADT) was 50% in the prior period, 40% in the middle period, and 30% in the latter period, respectively. The proportions of radiation therapy and active surveillance significantly increased. The proportion of radical prostatectomy remained similar over these periods (30%). The primary therapy was significantly different between the three periods (*p* < 0.001).

**Conclusions:**

High-risk patients significantly decreased in the latter period. The use of PADT also significantly decreased, while radiation therapy and active surveillance significantly increased over these periods.

## Background

Previous reports of the Japanese Urological Association indicated a distinctive trend in the use of primary androgen deprivation therapy (PADT) as the primary therapy for Japanese prostate cancer patients [1, 2]. We have also previously reported the trends in risk classification and primary therapy of patients with prostate cancer between 2004 and 2006 in the Nara Uro-Oncological Research Group (NUORG) registry. We found significant differences in the risk classification and primary therapy between Japanese and USA patients [3]. The proportion of high-risk patients was significantly higher in Japan than the USA, and the proportion of patients undergoing PADT was also significantly higher in Japan than the USA [1, 3–5]. Since our first report [3], we have also reported studies performed between 2004–2006 and 2007–2009 [6]. A dramatic decrease (10%) in PADT and increase (10%) in radiation therapy became apparent. We further investigated the changes in patient characteristics and primary therapy between 2010 and 2012 in the Nara Urological Research and Treatment Group (NURTG) (former: NUORG) registry, and compare these results with those of the previous survey performed between 2004 and 2009.

## Methods

A total of 7768 patients who were newly diagnosed with prostate cancer based on the NURTG registry (NURTG consists of Nara Medical University hospital and its 23 affiliated hospitals) between January 2004 and December 2012 were enrolled in this retrospective study. The clinical TNM classification (UICC 2002), biopsy Gleason score, prostate-specific antigen (PSA) at diagnosis and primary therapy were surveyed. We used the risk classification of the National Comprehensive Cancer Network (Version 1. 2013). Patients with cT1-2a N0 M0, PSA of <10 ng/mL at diagnosis and Gleason score of ≤6 were classified as “Very low to low” risk, those with T2b-c, PSA of 10–20 ng/mL, or Gleason score of 7 as “Intermediate” risk, those with T3a, Gleason score of 8-10, and PSA of >20 ng/mL as “High” risk, while those with cT3-4N0N0 were further defined as “Locally advanced” risk, and patients with node or distant metastases were defined as “metastatic.”

The baseline characteristics (stage, PSA distribution, age, Gleason score, and risk classification) between the prior (2004–2006), middle (2007–2009) and latter (2010–2012) periods were compared. Any differences in the primary therapy between the prior, middle, and latter periods were also compared.

To examine the differences in categorical parameters, the chi-square test was performed. The one-way ANOVA test was used to compare metric variables. All statistical analyses were performed using PASW Statistics 17.0 (SPSS Inc., Chicago, IL, USA). All *p* values of <0.05 were considered to be statistically significant.

The Medical Ethics Committee of Nara Medical University approved this retrospective study, and it was exempted from obtaining informed consent from the patients in consideration of the aim and methods of the study.

## Results

**Fig. 1:**
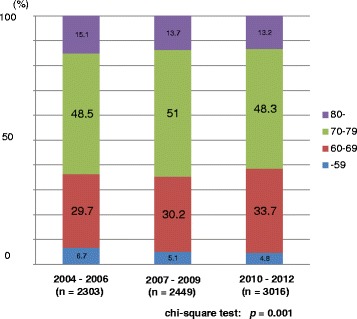
Distribution of age at diagnosis

**Fig. 2:**
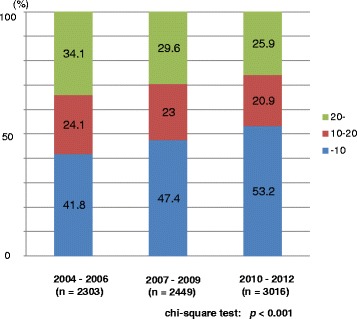
Distribution of the PSA value (ng/mL) at diagnosis

**Fig. 3:**
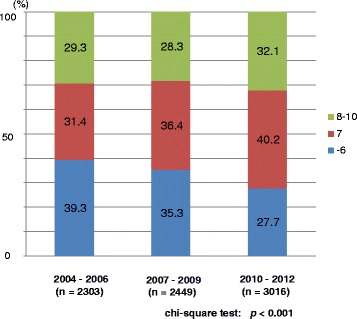
Distribution of the biopsy Gleason score

**Fig. 4:**
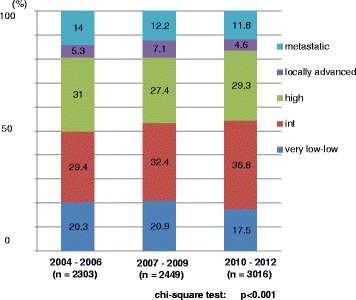
Distribution of risk classification of the NCCN

Of a total of 7768 patients, 2303, 2449, and 3016 patients were diagnosed between 2004–2006, 2007–2009, and 2010–2012, respectively. The mean (median) values of patients’ age were 71.8 (72.0), 71.9 (72.0), and 71.7 (72.0) years in the prior, middle, and latter periods, respectively. The mean (median) values of the PSA value at the time of diagnosis in the prior, middle and latter periods were 137.8 (12.2), 102.1 (10.8), and 99.6 (9.4) ng/mL, respectively. There was a significant difference in the PSA value at diagnosis among the 3 groups (*p* = 0.047, one-way ANOVA test). The demographic characteristics of all 7768 patients are shown in Table 1. The proportions of older patients (80 years or older) was significantly higher in the prior period whereas the proportion of middle-age (60–69 years) patients was significantly higher in the latter period (*p* = 0.001) (Fig. 1). The proportion of patients with a lower PSA value at diagnosis was significantly higher in the latter period (*p* < 0.001) (Fig. 2). The proportion of patients with a Gleason score of 7 was also significantly higher in the latter period (*p* < 0.001) (Fig. 3). The proportion of patients with T2 category significantly increased and that of T3 category decreased in the latter period (*p* = 0.014). There were no differences in the clinical N category distribution between the 3 periods, while the proportion of metastatic patients was significantly higher in the prior period (*p* = 0.003). Regarding risk classification, the proportions of high-risk and metastatic patients were significantly higher in the prior period, while the proportion of intermediate-risk patients was significantly higher in the latter period (*p* < 0.001) (Fig. 4). The risk classification was significantly different when comparing the prior and middle, middle and latter, and prior and latter periods, respectively.

**Table 1:** Demographic characteristics of 7768 patients

	Overall	2004–06	2007–09	2010–12	
	*n* = 7768 (%)	*n* = 2303 (%)	*n* = 2449 (%)	*N* = 3016 (%)	*P* value
*Age (years)*					
< 60	424 (5.5)	154 (6.7)	124 (5.1)	146 (4.8)	
60–69	2438 (31.4)	68.4 (29.7)	739 (30.2)	1015 (33.7)	
70–79	3825 (49.2)	1117 (48.5)	1250 (51.0)	1458 (48.3)	
≥ 80	1081 (13.9)	348 (15.1)	336 (13.7)	397 (13.2)	0.001
*PSA at diagnosis*					
10.0 or less	3729 (48.0)	963 (41.8)	1160 (47.4)	1606 (53.2)	
10.1–20	1746 (22.5)	554 (24.1)	563 (23.0)	629 (20.9)	<0.001
> 20	2293 (29.5)	786 (34.1)	726 (29.6)	781 (25.9)	
*Gleason score*					
-6	2606 (33.5)	906 (39.3)	865 (35.3)	835 (27.7)	
7	2827 (36.4)	722 (31.4)	892 (36.4)	1213 (40.2)	
8–10	2335 (30.1)	675 (29.3)	692 (28.3)	968 (32.1)	<0.001
*Clinical T stage*					
T1	2580 (33.2)	766 (33.3)	839 (34.3)	975 (32.3)	
T2	3260 (42.0)	933 (40.5)	986 (40.3)	1341 (44.5)	
T3	1544 (19.9)	489 (21.2)	489 (20.0)	566 (18.8)	
T4	384 (4.9)	115 (5.0)	135 (5.5)	134 (4.4)	0.014
*Clinical N stage*					
N0	7237 (93.2)	2161 (93.8)	2278 (93.0)	2798 (92.8)	
N1	531 (6.8)	142 (6.2)	171 (7.0)	218 (7.2)	0.296
*Clinical M stage*					
M0	6964 (89.6)	2019 (87.7)	2207 (90.1)	2738 (90.8)	
M1a	25 (0.3)	11 (0.5)	6 (0.2)	8 (0.3)	
M1b	719 (9.3)	257 (11.2)	211 (8.6)	251 (8.3)	
M1c	60 (0.3)	16 (0.7)	25 (1.0)	19 (0.6)	0.003
*Risk classification (NCCN)*					
Very Low to Low	1505 (19.4)	467 (20.3)	511 (20.9)	527 (17.5)	
Intermediate	2581 (33.2)	677 (29.4)	793 (32.4)	1111 (36.8)	
High	2269 (29.2)	715 (31.0)	670 (27.4)	884 (29.3)	
Locally advanced	435 (5.6)	121 (5.3)	175 (7.1)	139 (4.6)	
Metastatic	978 (12.6)	323 (14.0)	300 (12.2)	355 (11.8)	<0.001

Chi-square test

### Differences in primary therapy

**Fig. 5:**
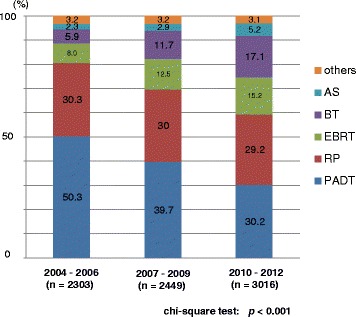
Distribution of the primary therapy of all 7768 patients (Chi-square test; *p* < 0.001). RP: radical prostatectomy, PADT: primary androgen deprivation therapy, EBRT: external beam radiation therapy, BT: brachytherapy, AS: active surveillance

Half of the patients received PADT in the prior period, while approximately 40% and 30% of patients received PADT in the middle and latter periods, respectively. The proportion of radical prostatectomy (RP) was the same (30%) in the three periods. The proportion of radiation therapy (RT), including both external beam radiation therapy (EBRT) and brachytherapy (BT), and active surveillance increased over the years. The primary therapy was thus significantly different between the prior and the latter periods (*p* < 0.001) (Fig. 5).

**Fig. 6:**
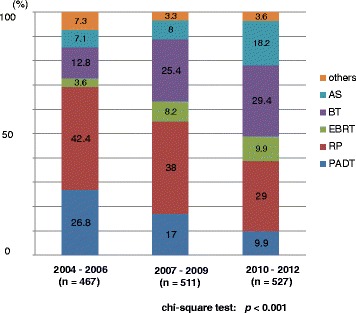
Distribution of the primary therapy of the very low- and low-risk patients (Chi-square test; *p* < 0.001). RP: radical prostatectomy, PADT: primary androgen deprivation therapy, EBRT: external beam radiation therapy, BT: brachytherapy, AS: active surveillance

**Fig. 7:**
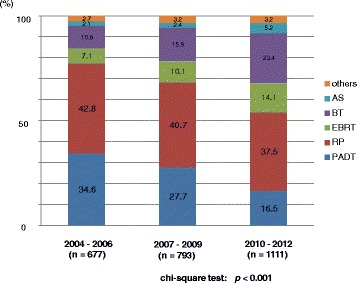
Distribution of the primary therapy of the intermediate-risk patients (Chi-square test; *p* = 0.013). RP: radical prostatectomy, PADT: primary androgen deprivation therapy, EBRT: external beam radiation therapy, BT: brachytherapy, AS: active surveillance

**Fig. 8:**
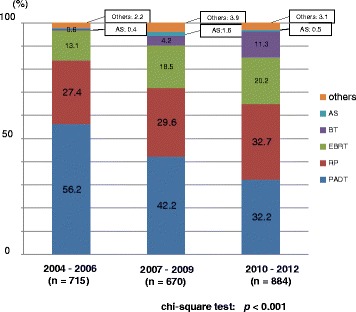
Distribution of the primary therapy of the high-risk patients (Chi-square test; *p* < 0.001). RP: radical prostatectomy, PADT: primary androgen deprivation therapy, EBRT: external beam radiation therapy, BT: brachytherapy, AS: active surveillance

**Fig. 9:**
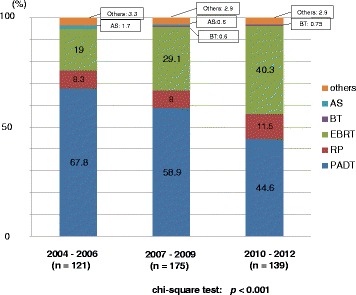
Distribution of the primary therapy of locally advanced patients (Chi-square test; *p* = 0.068). RP: radical prostatectomy, PADT: primary androgen deprivation therapy, EBRT: external beam radiation therapy, BT: brachytherapy, AS: active surveillance

The primary therapy of very low- to low- and intermediate-risk patients showed a significant increase in active surveillance over the years (Figs. 6, 7). The proportion of EBRT and BT significantly increased in the very low-to low-, intermediate-, and high-risk patients (Figs. 6, 7 and 8). The proportion of EBRT also significantly increased in the locally advanced patients (Fig. 9). The proportion of PADT significantly decreased in all non-metastatic patients, while that of RT significantly increased over the years (Figs. 6, 7, 8 and 9). The primary therapy was significantly different when comparing the prior and middle, middle and latter, and prior and latter periods, respectively, except for “locally advanced” and “metastatic” in the prior and middle periods, and “metastatic” in the middle and latter periods.

## Discussion

In Japan, the age-adjusted incidence rate of prostate cancer have shown an increasing trend, while the age-adjusted mortality rate have shown a decreasing trend year after year as reported by the National Cancer Center Japan. The projected incidence and number of deaths due to prostate cancer take 1st and 6th place, respectively, among all cancers for Japanese men in 2016 (http://gdb.ganjoho.jp). We have previously reported the trends in risk classification and primary therapy of patients who had been diagnosed with prostate cancer in the Nara Uro-oncological Research Group registry between 2004 and 2006 [3]. Approximately 50% of the patients showed high-risk features and received PADT according to this report. This result was compatible with reports by the Japanese Urological Association (JUA) [1, 2] that 57% and 50% of patients received PADT in 2000 and 2004, respectively. The proportion of high-risk patients and the use of PADT in Japan were significantly higher than in the USA [3, 4, 7, 8].

We also reported the changes in the trends in risk classification and primary therapy in the NUORG data registry between the 2004–2006 and 2007–2009 periods [6]. The proportion of high-risk patients decreased significantly, and the proportion of PADT also decreased significantly from 50% to 40% during this period. Onozawa et al. also reported that the proportion of PADT in 2010 was 40% in a multi-institutional observational study by the Japan Prostate cancer Study Group (J-CaP study) [9].

As we have previously reported, the proportion of very low- and low- risk patients did not increase, while the proportion of intermediate-risk patients significantly increased from 2004 to 2006 to 2010–2012. The migration in the risk classification to intermediate risk by the introduction of the Gleason grading proposed by the 2005 International Society of Urologic Pathology (ISUP) Gleason Grading Consensus [10] was the conceivable reason for this trend. All institutes involved in the present study have introduced the ISUP (2005) Gleason Grading Consensus to make a diagnosis of prostatic cancer in 2006. Indeed, the proportion of patients with a higher PSA value at diagnosis significantly decreased, while the proportion of patients with a Gleason score of 7 significantly increased over these periods (Table 1, Fig. 3). Although the exact PSA exposure rate of Japanese men has never been surveyed, the low PSA exposure rate of Japan compared with the USA also influenced the difference in risk classification as we have mentioned in the previous study [3]. Indeed more than 45% of patients still have a high risk or worse in the latter period (2010–2012) (Fig. 4). On the other hand, it can be seen that the proportion of patients who underwent active surveillance and definitive treatment among patients with localized and locally advanced disease steadily increased.

The present study demonstrates an especially pronounced decreasing trend in PADT in the latter 3 years (2010–2012). The proportion of PADT significantly decreased from 50% (2004–2006) and 40% (2007–2009) to 30% (2010–2012). The proportion of active surveillance also significantly increased from 2.3% (2004–2006) and 2.9% (2007–2009) to 5.2% (2010–2012) (Fig. 5). Regarding definitive treatment (RP and RT), the proportion of RT significantly increased from 13.9% (EBRT: 8.0%, BT: 5.9%) (2004–2006) and 24.2% (EBRT: 12.5%, BT: 11.7%) (2007–2009) to 32.3% (EBRT: 15.2%, BT: 17.1%) (2010–2012), while the proportion of RP remained constant (30%) during this period.

In Japan, low-dose-rate BT was approved in 2003. We also started low-dose-rate BT in 2004 [11]. Almost all patients in this study received low-dose-rate BT. We performed low-dose-rate BT not only for low-risk patients, but also for intermediate- and high-risk patients in combination with EBRT and androgen deprivation therapy. Coincidently, intensity modulated radiation therapy (IMRT) has come to be widely used. Indeed, the present study revealed that the proportion of RT (EBRT and RT) has significantly increased over these periods. The favorable oncologic outcome of radiation therapy has been recognized during the last decade [12, 13]. In the prior period (2004–2006), physicians were apt to choose PADT if patients refused to undergo RP [6]. This trend could be seen at hospitals where definitive radiation therapy was not available [14]. The recognition and realization of the usefulness and efficacy of definitive radiation therapy as well as RP led a variety of primary therapy choices for general urologists. Indeed, a decrease in PADT and an increase in RT can be seen for all risk groups in the present study (Figs. 6, 7, 8 and 9).

## Conclusion

A significant migration in the risk classification toward intermediate risk could be seen in Japanese prostate cancer patients between the prior (2004–2006) to the middle (2007–2009) and latter (2010–2012) periods. However, patients still had a higher risk than in the USA. The primary therapy also changed over these years. The use of PADT significantly decreased and the proportion of active surveillance and radiation therapy increased, not only in the overall population, but also in each risk group separately.

## Abbreviations

BT: brachytherapy
EBRT: external beam radiation therapy
IMRT: intensity modulated radiation therapy
NUORG: Nara Uro-Oncological Research Group
NURTG: Nara Urological Research and Treatment Group
PADT: primary androgen deprivation therapy
PSA: prostate-specific antigen
RP: radical prostatectomy
RT: radiation therapy
